# Current Status of the Diagnosis and Management of Osteoporosis

**DOI:** 10.3390/ijms23169465

**Published:** 2022-08-21

**Authors:** Agustín Aibar-Almazán, Ana Voltes-Martínez, Yolanda Castellote-Caballero, Diego Fernando Afanador-Restrepo, María del Carmen Carcelén-Fraile, Elena López-Ruiz

**Affiliations:** 1 Department of Health Sciences, Campus de Las Lagunillas SN, University of Jaén, E-23071 Jaén, Spain; 2 Centre for Biomedical Research (CIBM), Biopathology and Regenerative Medicine Institute (IBIMER), University of Granada, E-18011 Granada, Spain; 3 Biosanitary Research Institute of Granada (ibs.GRANADA), University Hospitals of Granada, University of Granada, E-18014 Granada, Spain; 4 BioFab i3D-Biofabrication and 3D (Bio) Printing Laboratory, University of Granada, E-18011 Granada, Spain; 5 ZIPATEFI Research Group, Faculty of Health Sciences and Sports, University Foundation of the Área Andina, Pereira 660001, Colombia

**Keywords:** osteoporosis, regenerative medicine, lifestyle habits

## Abstract

Osteoporosis has been defined as the silent disease of the 21st century, becoming a public health risk due to its severity, chronicity and progression and affecting mainly postmenopausal women and older adults. Osteoporosis is characterized by an imbalance between bone resorption and bone production. It is diagnosed through different methods such as bone densitometry and dual X-rays. The treatment of this pathology focuses on different aspects. On the one hand, pharmacological treatments are characterized by the use of anti-resorptive drugs, as well as emerging regenerative medicine treatments such as cell therapies and the use of bioactive hydrogels. On the other hand, non-pharmacological treatments are associated with lifestyle habits that should be incorporated, such as physical activity, diet and the cessation of harmful habits such as a high consumption of alcohol or smoking. This review seeks to provide an overview of the theoretical basis in relation to bone biology, the existing methods for diagnosis and the treatments of osteoporosis, including the development of new strategies.

## 1. Introduction

In 1993, the WHO defined osteoporosis as a systemic skeletal disease characterized by low bone mass, the deterioration of the microarchitecture of bone tissue, a consequent increase in bone fragility and a susceptibility to fractures [1]. In addition, osteoporosis has been reported to occur when there is an imbalance in bone cell function [2]. This disease has been called “the silent epidemic of the 21st century” because of its public health implications. It is a severe, chronic, progressive and clinically silent disease and the most common of the metabolic bone diseases [3].

Within osteoporosis, there are several types that can be classified into two large groups: primary and secondary osteoporosis. Primary osteoporosis includes idiopathic osteoporosis occurring in children and young adults, with an unknown etiopathogenesis [4], and involutional osteoporosis affects both men and women and is more related to aging [5]. Likewise, involutional osteoporosis is divided into type I or postmenopausal osteoporosis, which mainly affects women between 51 and 75 years of age and is characterized by rapid bone loss [6]. Type II or senile osteoporosis occurs in persons over 75 years of age and is characterized by a loss of trabecular and cortical bone that results from aging [3]. Secondary osteoporosis accounts for less than 5% of all cases of osteoporosis and is a consequence of a disease or the use of medications [7]. Among all of them, the most frequent kind of osteoporosis is postmenopausal osteoporosis, which is linked to two conditions: menopause and aging [6].

Among the metabolic bone diseases known to date, osteoporosis is not only the most frequent but is also a major global public health problem due to its high morbidity, which is caused by osteoporotic fractures in the older population [8]. This process occurs in people of both sexes and in the different types of osteoporosis, and it is also known to affect both pediatric and young patients, either primary or secondary to systemic diseases or medical treatments [9]. The National Institute Health Consensus on Prevention, Diagnosis and Therapy of Osteoporosis concluded that “bone mass acquired early in life may be the major determinant of long-term bone health” [10].

Due to the fact that bone loss is produced by advancing age, the prevalence of osteoporosis increases with it; therefore, as a chronic and prolonged skeletal disorder, it is more common in senile people, occurring in men over 65 years of age and in women over 55 years of age, approximately [11]. However, in women, it is more frequent due to other symptoms produced by menopause. During this stage, the estrogen deficit produces an increase in bone remodeling, which causes the loss of bone density [12]. In fact, in 2010, it was observed that 5.5 million men and 22 million women in the European Union had osteoporosis according to the diagnostic criteria used by the WHO [13], with 80% of the female population being unaware of the risk factors before being diagnosed with the disease [14].

Osteoporosis does not follow pre-established clinical patterns and manifests itself in various ways during its course. Individuals with uncomplicated osteoporosis may remain asymptomatic until a fracture occurs [5]. Although osteoporosis presents a general symptomatology, it also manifests with specific signs and symptoms such as: (i) pain that is secondary to osteoporotic fractures, which can occur in any bone and whose clinical manifestations depend on the location [15]; (ii) deformities and multiple vertebral compression fractures which can produce an increase in thoracic kyphosis and cervical lordosis [16]. The last ribs could contact the iliac crest, causing the relaxation of the diaphragm, which is the cause of digestive (hiatus hernia, meteorism) and respiratory (dyspnea) manifestations [17]. Moreover, there are alterations of the adipose panniculus and the presence of skin folds on the back, pubic region and umbilicus [18]. Likewise, hyperkyphosis causes cervical pain as the patient tries to keep the head upright through cervical hyperextension [19]. Moreover, increased dorsal kyphosis also occurs in osteoporotic males, resulting in shoulder droop, compensatory lumbar, cervical hyperlordosis and a characteristic postural habitus [20]. (iii) A loss of height, as vertebral fractures and hyperkyphosis can result in a decrease in height of about 10–20 cm, approximately [21].

Many factors are involved in the development of osteoporosis. Some of them are modifiable, such as environmental factors and some endocrine factors. Environmental factors include: (a) nutritional factors, such as deficient calcium intake, vitamin D deficiency due to nutritional problems, poor absorption or low sun exposure, excessive protein intake in unbalanced diets, excessive phosphate intake or excessive salt intake that increases urinary calcium loss [22]; (b) sedentary lifestyles, anaerobic exercise and excessive mechanical load, which are three factors that directly cause the risk of osteoporosis [23]; (c) chronic pharmacological treatment such as anti-convulsants, glucocorticoids, sedatives or chemotherapy; (d) the intake of caffeine, alcohol or smoking [24]; (e) body weight, which is responsible for 15% to 30% of the variations in bone mineral density (BMD) at any age and in any measured bone region [25]. Endocrine factors include: (a) late menarche or menstrual cycle alterations, which are conditions that are associated with low bone mass [26]; (b) surgical or non-surgical menopause before the age of 45 years [27]; (c) being a hormonally infertile woman [28]; and (d) estrogen deficiency before menopause as a result of anovulation due to anorexia nervosa, excessive exercise, mental stress, etc. This is the most important risk factor for osteoporosis, at least in Western countries [29]. It is important to look at these modifiable factors because they could be corrected and decrease the risk of developing osteoporosis [30].

In addition, there are non-modifiable risk factors such as genetics, since there are important genetic components in the determination of bone density and mass [31], e.g., race, since Caucasians and Asians are at a greater risk than Blacks and Polynesians [32]; sex, since it has been found that the risk is greater in women than in men [33]; and age, since each decade increases the risk by 1.4 to 1.8 times. It is another clear cause of bone density loss, not only because of the drop in hormone levels but also because, histologically, there is a decrease in the average thickness of the bone wall, but bone resorption remains high with aging [34].

Due to the increase in life expectancy produced by the aging of the world population, osteoporosis is becoming an emerging health problem, representing one of the main non-communicable diseases at this time, and it can interfere negatively in the quality of life of people. Therefore, it is essential to know the factors involved in this disease and to establish approaches for its management and treatment [35].

## 2. Bone Biology

Bone tissue is a dynamic, mineralized connective tissue that serves multiple physiological functions [36]. Bone provides mechanical support for loading and locomotion, offers physical protection to internal soft organs, forms a non-static reservoir of calcium and phosphate ions and provides an environmental niche for bone marrow and hematopoietic cell development.

In bone, there is a hierarchical structure with two separate phases: the organic matrix and the inorganic matrix [37]. The organic matrix is composed mainly of type I collagen, the fibers of which are linked by triple helix cross-links. It is this structure that provides the bone with resistance to longitudinal tensile forces as well as elasticity. On the other hand, the inorganic matrix is mineralized with hydroxyapatite and calcium phosphate crystals, which are located in the free voids of the organic matrix. This matrix is responsible for the stiffness of the bone and its resistance to compressive forces in a way that depends on the amount of mineral, the arrangement of the crystals and the degree of packing [38].

The remaining bone volume is composed of bone cells of two classes: osteoprogenitor cells and osteoclasts. Osteoprogenitor cells are derived from mesenchymal stem cells (MSCs) that subsequently differentiate into osteoblasts and osteocytes. The differentiation of these cells is initiated when they receive migration signals to a certain area, proliferate and, finally, differentiate. Osteoblasts are the cells that line the surfaces of bone and are responsible for the synthesis and secretion of the organic bone matrix. Osteocytes are the majority of bone cells capable of communicating directly with each other [39,40]. All these cells are responsible for maintaining the bone matrix and regulating calcium homeostasis, although they also play an important role in bone resorption. Finally, osteoclasts are the largest cells, have multiple nuclei and are of hematopoietic origin. They are bone resorption cells and act by phagocytosing the matrix through acidification solubilization [41,42].

Furthermore, bone tissue can be differentiated into cortical bone or trabecular bone. Both types of bone are similar in their cellular and molecular composition but different in terms of functionality and mechanical characteristics [43]. Cortical bone is the bone found in the outermost part of the long bones. It is a very compact tissue that circulates the blood vessels, the canaliculi that surround the osteocytes and their connecting cellular processes. On the contrary, trabecular bone, also called cancellous bone, is found in the epiphysis of long bones, in the vertebrae and near the articular surfaces. It consists of a network of thin bony plates and connecting struts surrounded by the bone marrow [44].

**Figure 1:**
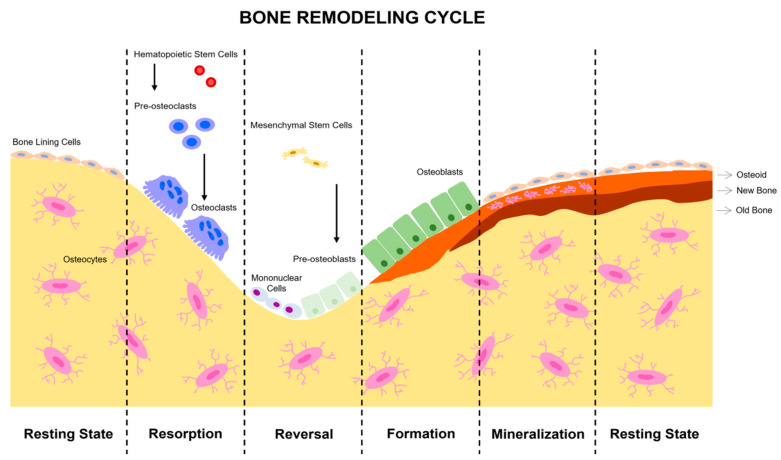
Stages of bone remodeling. In a balanced system, bone remodeling begins with bone resorption and ends with osteoblast formation. The complete cycle is composed of the phases of activation, resorption, reversion, formation and, finally, mineralization. Initially, a signal is detected which activates resorption by attracting osteoclast precursors to the area to be remodeled. This phase is of limited duration and depends on the degree of stimuli received, causing osteoclast differentiation and activity. Then, in the reversion phase, almost all of the osteoclasts disappear, and osteoblast precursors of mesenchymal origin begin to form. In the formation phase, all the osteoclasts are definitively replaced by osteoblasts. Finally, the mineralization of new bone tissue occurs. The new tissue remains at rest until the next cycle of remodeling.

Bone remodeling begins in fetal life and continues throughout our lives, adapting the shape of bones by removing and adding bone tissue at different key points [45]. Bone remodeling is crucial for the repair of bone damaged by constant physical loading and the prevention of fractures of various origins. This process is based on the balance of two main phases: bone formation and bone resorption (Figure 1) [46]. Bone is unique in the healing of connective tissue because it is capable of complete healing through cell regeneration and mineral matrix production [47]. As mentioned above, bone tissue is in a constant process of remodeling, which allows the skeleton to renew itself continuously. This remodeling process is directly related to mechanical stresses. This can prevent excessive fatigue damage, ensure the viability of bone cells, repair microfractures or allow for proper calcium homeostasis. The constant changes in bone mass and architecture due to load-bearing are regulated by osteoclasts together with the osteoblast–osteocyte communication system [48]. These bone cells form the main mechanical sensor network of the tissue.

During the remodeling process, there are several markers by which we can identify the existence of bone formation. Some of these markers are indicators of osteoblastic activity and the resulting metabolism after collagen release [49]. Alkaline phosphatase (ALP) is an enzyme associated with the plasma membrane of cells produced by osteoblasts, which play an important role in osteoid formation and mineralization. Its absence can lead to the development of liver disease. Osteopontin (OPN) is another non-collagenous protein with a key role in the structure and mechanics of bone tissue. A charged and phosphorylated protein with a high affinity for calcium, it has been attributed multiple functional roles in bone mineral bioregulation. It acts as a link at the mineral–collagen interface, improving bone hardness [50]. Another useful marker is osteocalcin (OC). OC is the most abundant non-collagenous protein present in the bone matrix. It is a small hydroxyapatite-binding protein synthesized by osteoblasts. It is generated during bone formation and can be released during bone resorption. This protein is rapidly degraded in vivo and ex vivo. Collagen type I is a protein synthesized by the osteoblasts and also serves as a bone formation marker [51].

Disuse can lead to deterioration in bone density and architecture, but physical exercise can slow the progression of these problems [52]. The mechanical forces supported by the cells in this tissue type are complex and multifactorial systems. The response of cells to these forces is regulated by cytoskeletal proteins and transmembrane-bound integrins that link the extracellular microenvironment with the genetic load in the nucleus. The bone marrow is also indirectly involved in bone remodeling. It produces MSCs that are also subjected to these loads, along with dynamic shear forces derived from the bone marrow bone interface. It is precisely these forces that promote osteogenesis and the cell differentiation of MSCs into an osteoblast lineage by dynamically activating the actin structure in the cytoskeleton while inhibiting adipogenesis [53].

Over the years, bones become more fragile and lose their functionality [54]. Factors such as immobilization, hormonal or nutritional deficiencies or chronic diseases can metabolically affect bone remodeling leading to osteopenia [55]. Therefore, the regulation of cellular and molecular processes to maintain the balance between bone resorption and bone formation is fundamental. An imbalance in this process can lead to the loss of bone density and mineral homeostasis, resulting in osteoporosis [56]. Osteoclasts, osteoblasts and osteocytes are bone cells directly involved in bone remodeling and a failure in their molecular mechanism is the possible trigger of the disease [57,58].

Several hormones, primarily estrogens [59], are responsible for regulating bone remodeling by controlling the cytokines and growth factors produced by bone marrow and bone cells. However, bone remodeling is also regulated by other systemic regulators (parathyroid hormone (PTH), vitamin D, calcitonin or glucocorticoids) and local regulators such as cytokines, growth factors mainly transforming growth factor beta (TGF-β), the macrophage colony-stimulating factor (M-CSF), receptor activators of nuclear factor-κB ligand (RANKL) or prostaglandins [58,60].

It is well known that the loss of estrogens increases bone resorption in women and, to some extent, in men. This is only supered by age-related osteoporosis. There are various factors, such as the low absorption of calcium and vitamin D and aging, which cause a decrease in the production of estrogens. The main cause of osteoporosis in women is menopause due to sex hormones reduction. A low production of estrogens causes the prolonged maintenance of osteoclasts. while osteoblastic cells deteriorate, leading to a homeostatic imbalance of the bone [61].

The action of these systemic regulators such as vitamin D and calcium exchange is essential for the physical resistance of bone and is closely related to PTH, one of most prominent regulatory hormones. Vitamin D levels are inversely related to PTH; if existing vitamin D decreases, PTH increases. This leads to a negative calcium balance and, consequently, to the deterioration of bone tissue [62].

Functional PTH receptors are found in osteoblasts, regardless of their maturation state. Problems in PTH regulation, such as ongoing hyperparathyroidism, result in a severe loss of bone mass, even though bone formation by osteoblasts continues. Although it is known that PTH plays a fundamental role in bone remodeling, it is not possible to determine how it is able to promote bone formation, since there is no single mechanism that explains it but rather multiple complementary mechanisms that act in a coordinated manner [63]. PTH anabolic treatments were the first Food and Drug Administration (FDA)-approved osteoporosis medications that could stimulate new bone formation [64].

On the other hand, the main signaling pathways controlling osteoclastic bone resorption and osteoblastic bone formation are the receptor activators of nuclear factor-κB (RANK)/RANKL/osteoprotegerin (OPG) and the canonical Wnt signaling [65,66].

First, for the initiation of the RANKL/RANK/OPG signaling pathway to occur, there must be an adequate concentration of M-CSF, which is expressed by osteocytes and osteoblasts. It stimulates the expression of RANK necessary before the action of RANKL. Subsequently, the binding of RANKL to its receptor on osteoclast precursor cells drives osteoclast differentiation, facilitating their activation and survival. RANKL/RANK binding induces a cascade of protein signaling molecules to enable osteoclast gene expression. RANKL produced by osteocytes is thought to sense changes in tissue load and initiate the bone remodeling cycle by stimulating osteoclastogenesis. Finally, OPG is a RANK receptor secreted by osteoblasts and osteocytes capable of inhibiting osteoclastic bone resorption by binding to RANKL instead of RANK [56]. The other key signaling pathway, the canonical Wnt pathway, is dependent on β-catenin, an important regulator of osteoblastic bone formation [65]. In the absence of Wnt, the cytoplasmic β-catenin glycoprotein is marked by proteasomal degradation that phosphorylates and ubiquitinates β-catenin. Because of this, the expression of the Wnt target gene is inhibited. If Wnt is present, it binds to a dual receptor complex comprising the Frizzled family of proteins, a seven transmembrane domain receptor and a lipoprotein-related co-receptor (LPL) (5 or 6). This blocks the action of the destruction complex, leading to the accumulation of cytoplasmic β-catenin, which ultimately promotes osteoblast proliferation and differentiation [56].

## 3. Diagnosis of Osteoporosis and Fracture Risk Assessment

Nowadays. the diagnosis of osteoporosis is mainly based on the evaluation of bone mass by bone densitometry (DEXA) [67]. Although osteoporosis is more than a bone densitometry value, this evaluation allows for the quantification of bone tissue, which is used as a diagnostic criterion and is considered a predictive value for the risk of fracture, which makes it the best method for determining the rate of bone loss and as a reference point for the evolutionary control of the disease [68].

According to the WHO Expert Committee, the classification of BMD values is as follows: (i) normal: BMD > −1 SD t-score; (ii) osteopenia: BMD between −1 SD and −2.5 SD t-score; (iii) osteoporosis: BMD < −2.5 SD t-score; and (iv) established osteoporosis: BMD < −2.5 SD t-score + fragility fracture [69]. The T-score or t-value, which is the number of standard deviations above or below the mean BMD of the normal young population of the same sex, has been taken into account for this classification [70]. However, in the case of premenopausal women, men under 50 years of age and children, the Z-score will be considered (in relation to normal subjects of the same age and sex) such that “normal” will be considered up to −2.0 [71]. This classification is, to date, universally accepted as a diagnostic criterion. Its sensitivity and specificity are close to 90%, and it may be able to increase the detection of patients who would not be classified as osteoporotic. However, limitations exist in this imaging test, especially in the presence of osteomalacia, osteoarthrosis and osteoarthritis [72]. In Europe, the International Osteoporosis Foundation (IOF) has carried out a campaign where diagnostic bone density tests (densitometry) were performed in people at an increased risk of the disease [5]. The results of this campaign in Spain were worrisome: of the 900 citizens in an age group between 50 and 70 years who underwent densitometry tests, about 25% suffered from the disease, and approximately the same percentage had osteopenia, a degree of bone degeneration. In this campaign, it was also found that most of the citizens who underwent densitometry had done for the first time [73]. There are also several diagnostic tests used to monitor the treatment of osteoporosis in clinical practice [74], including dual X-ray absorptiometry, which is the most recommended technique for the diagnosis of osteoporosis since it can predict the risk of fracture, indicate the treatment or monitor its effect [75]. Dual X-ray absorptiometry is based on the quantification of axial bone mineral density (spine and hip) by measuring the transmission of a beam of X-ray photons with two energy peaks in the patient’s body, which allows for the assessment of the calcium content of the bone [76]. A study conducted in postmenopausal women showed that BMD and fracture risk were related, thus defining osteoporosis as a t-score value of −2.5 [77].

On the other hand, general blood and urine tests provide information on the general health status and on the existence of elements causing secondary osteoporosis [76]. These markers are really useful tools in identifying metabolic bone diseases, since they provide us with information that is not directly obtained with a bone density measurement or bone histomorphometry [78]. With respect to markers, another commonly used test is bone turnover markers (BMTs), which are capable of measuring peptides of the amino and carboxy-terminal ends in processes of bone matrix formation or degradation [79]. Among these are formation markers that measure osteoblastic activity, i.e., bone-forming activity, such as ALP and OC. ALP is secreted by different tissues (liver, bone, placenta), and its most frequent isoforms are from hepatic and bone (90%) [80]. The bone isoform does not vary between sexes and is not influenced by the circadian rhythm, which makes it a simple marker, although it has a low sensitivity and specificity in metabolic bone disorders [81]. In situations of increased bone turnover, the half-life of OC decreases, and it is eliminated through urine [82]. On the other hand, the most commonly used resorption markers that measure osteoclast activity are: (i) Pyridinolines (Pir) and deoxypyridinoline (Dpir), which link collagen molecules in the bone matrix through covalent bonds, thus forming fibrils [83]; and (ii) ICTP (C-terminal telopeptide of type I collagen), β-CTX (β-CrossLaps) and NTX (N-terminal telopeptide of type I collagen), which are peptides released during the process of bone resorption. β-CTX and NTX are considered to be the most useful resorption markers in clinical practice for the diagnosis of osteoporosis [78].

In addition, there are other methods, such as ultrasound based on measuring sound velocity and ultrasound attenuation in peripheral skeletal bones. However, it has not been demonstrated that the parameters obtained by this test are clinically useful for monitoring the disease; another assessment technique is quantitative computed tomography, which is based on the measurement of BMD volume in trabecular and cortical bones; however, this is a tool that is not recommended, since its economic cost is very high, and it exposes the patient to greater ionizing radiation than DEXA [84]. Finally, osteoporosis could be diagnosed through a biopsy of bone tissue. This is a very invasive technique in which a tissue sample is extracted, and it is only performed when evidence of tumors is detected [85].

Fragility fractures are the most common consequence of osteoporosis and are particularly common in the vertebrae, hip and forearm. These fractures increase exponentially with age and are a major cause of morbidity and mortality in elderly populations [86]. Moreover, the proximal ends of the femur and humerus, the distal end of the radius and the spine are the most susceptible to osteoporotic fractures in comparison to other parts of the bone [87]. Likewise, hip fracture is considered to be the severe complication that is most associated with high morbidity and mortality [88].

Therefore, it is essential to assess the risk of fracture, which is performed by considering the degree of osteoporosis obtained by densitometry according to the WHO Expert Committee: (i) normal value: the risk of fracture is normal; (ii) osteopenia value: the risk of fracture is double the normal risk; (iii) osteoporosis value: the risk of fracture is quadruple the normal risk; (iv) established osteoporosis value: the risk of fracture for each reduced standard deviation is multiplied by 1.5–2; and (v) severe osteoporosis value: the risk is similar to that of established osteoporosis [89].

## 4. Treatment of Osteoporosis and Novel Approaches

### 4.1. Overview of Existing Drug Therapies and New Drug Development

Great advances have been made in the study of the pathogenesis of osteoporosis and in the development of new drugs for its treatment. The primary purpose of a pharmacological treatment is to reduce the risk of fractures and to improve the quality of life of people with osteoporosis [90]. It has been proven that, before beginning pharmacological treatment, it should be ensured that the person has adequate levels of both calcium and vitamin D, since the combination of these two elements has shown great synergy in promoting calcium absorption and in helping to maintain adequate serum calcium concentrations for the proper mineralization of the bone [91]. In the majority of cases, the calcium and vitamin D intake is insufficient in the diet; therefore, a supplement is always prescribed to achieve the recommended levels. Although some authors do not support the use of these supplements because of the adverse effects they could have, such as constipation, this is why their use is mainly recommended in postmenopausal women who do not meet the recommended levels through diet [92].

The drugs used for the treatment of osteoporosis can be divided according to the effect they produce on the bone. On the one hand, there are the antiresorptive drugs classified as ‘‘bone resorption inhibitors’’, and on the other hand, there are the anabolic agents classified as ‘‘bone formation accelerators’’ (Table 1) [93]. Mainly antiresorptive and anabolic drugs approved by the Food and Drug Administration (FDA) [94] are shown at Table 1.

These antiresorptive drugs suppress osteoclastogenesis and result in the suppression of bone turnover, thereby increasing mineralization. The function of anti-resorptive drugs is to decrease or prevent bone resorption by trying to balance the bone formation and bone resorption suppressing osteoclast function. Most of the treatments used in osteoporosis fall into this group: bisphosphonates, selective oestrogen receptor modulators (SERMs), calcitonin and denosumab [95]. On the other hand, anabolic agents increase bone turnover, which mostly affects bone formation. However, it has been suggested that the prolonged use of PTH analogs increases the risk of osteosclerosis and osteosarcoma due to their stimulatory effects, and they are not used for the long-term treatment of osteoporosis [93]. Among the most commonly studied anti-resorptive drugs are SERMs, which include raloxifene and azedoxifene. Raloxifene is capable of alleviating climacteric symptoms, preventing bone loss and increasing bone mineral density. Like other SERMs, Raloxifene can bind to the estrogen receptor (ER) in an agonistic or antagonistic manner depending on the target tissue [96]. Bazedoxifene, binds at the cellular level to both ERα and Erβ and inhibits its binding to 17β-estradiol, exerting agonistic activity on the bone [97].

Calcitonin is a thyroid hormone that binds at the calcitonin receptor expressed in the kidney, the hypothalamus and in the membranes of osteoclasts. Calcitonin works by inhibiting osteoclasts and reducing the bone resorption ability. Additionally, it can reduce fracture-related pain, apparently through regulating nociception in the central nervous system. It has a positive impact on reducing vertebral fractures in postmenopausal osteoporotic women. However, the treatment with calcitonin has declined over the years because the use of hormone therapy increases the risk of cardiovascular complications and breast cancer [98].

The most common first-line treatments for osteoporosis are bisphosphonates. Alendronate, or alendronic acid, is a nitrogen-containing bisphosphonate that binds to bone surfaces and inhibits bone resorption by osteoclasts, possibly by inhibiting the mevalonate pathway. It has been shown to be effective in the treatment of women or men with corticosteroid-induced osteoporosis and in the prevention of osteoporosis in postmenopausal women [99]. Risedronate is a pyridinyl bisphosphonate capable of reducing the bone turnover process and decreasing bone resorption by interfering with the activity of osteoclasts and inhibiting their adhesion to the mineralized bone matrix without affecting its porosity [100]. Another nitrogenous bisphosphonate is ibandronate, which has one of the best anti-resorptive capabilities due to the tertiary nitrogenous group on its R2 side chain and the hydroxyl group on its R1 side chain. It reverses the bone loss associated with estrogen depletion and has a strong binding affinity to hydroxyapatite [101]. On the other hand, zoledronic acid, a potent intravenous amino bisphosphonate, is an antiresorptive agent that improves bone mineral density, reduces fracture risk and bone turnover and maintains bone structure. It has a high affinity for mineralized bone and is primarily targeted at sites of increased bone turnover. In addition, it affects the endocytic activity of osteoclasts and inhibits bone resorption by inhibiting farnesyl pyrophosphate synthase (FPPS), which prevents protein prenylation [102].

Another popular medication used for treating osteoporosis is denosumab, a human monoclonal antibody targeting RANKL. RANKL is a protein essential for osteoclast formation, differentiation and survival and is a key mediator of bone resorption. Denosumab is more effective at improving bone density and strength than bisphosphonate drugs, but unlike bisphosphonate drugs, it is not incorporated into bone, so its effect ceases when the treatment is stopped [103].

Anabolic agents such as PTH analogs are also used in the treatment of osteoporosis, although to a lesser extent and generally when antiresorptive treatments are not effective. Currently, only two osteoanabolic drugs are available for the treatment of osteoporosis: Teriparatide and Abaloparatide. Teriparatide, or human PTH (1-34) hormone, is a bone formation promoter derived from the PTH (1-84) chain, the main regulator of calcium formation and bone metabolism in mammals. The primary sequence of Teriparatide is identical to the 34 amino acids of full-length PTH (1-84), which correspond to the active phase of mineral homeostasis. Teriparatide promotes osteoblastogenesis and prevents osteoblastic apoptosis [104,105]. On the other hand, Abaloparatide is a 34-amino acid synthetic analog of PTH that is identical to that of the PTH peptide in the first 20 amino-acids. Abaloparatide promotes bone formation and has similar effects to Teriparatide. Some studies have shown that the use of Abaloparatide reduces the risk of vertebral fractures in postmenopausal women with osteoporosis compared to patients treated using alendronate [106]. Both Abaloparatide and Teriparatide treatments were furthermore able to generate an increase in RANKL and M-CSF mRNA expression in a human osteoblast line [107].

Moreover, new targets are being studied for the treatment of osteoporosis, such as cathepsin K inhibitors, which have been shown to cause a decrease in bone resorption, thus preserving bone, or antisclerostin therapies, products of the SOST gene which bind to the LRP5 or LRP6 receptors in such a way that they inhibit osteoblastic activity, promoting its apoptosis. Additionally, antibodies against sclerostin, an osteocyte-secreted protein that inhibits bone formation by inhibiting the Wnt signaling pathway by binding to the LRP5/6co-receptors, are in clinical development. Romosozumab is a sclerostin inhibitor approved by the FDA for the treatment of osteoporosis in postmenopausal women at a high risk of fracture that can act with both antiresorptive and osteoanabolic functions [108]. However, these novel osteoporosis therapies are still under study [109,110], and the current ones are not fully effective in all patients and also present serious side effects which limit their long-term use.

Therefore, there is an increasing need for the development of new therapies that base their mechanisms on bone biology, have no side effects and promote bone formation and thereby a reduction in the risk of fractures [111].

### 4.2. The Treatment Gap in Osteoporosis

Despite the epidemiological impact of osteoporosis, not all patients at a high risk of fractures are effectively assessed and treated with osteoporosis drugs [112]. This may be due to most high-risk individuals not being identified, not receiving appropriate treatment or, even when treated, not taking it. Hence, there is the so-called “treatment gap” in bone fragility management, which leads to an increase in the burden of osteoporotic fracture for individuals, societies and healthcare systems [113].

Although some clinical calculators, such as FRAX, are recommended by several guidelines for screening decisions, fracture risk calculation is still underestimated [114]. For example, the FRAX and Garvan risk calculators have demonstrated a low ability to identify the risk of hip fractures, major osteoporotic fractures or any clinical fractures in postmenopausal women aged 50–64 years during 10 years of follow-up [115].

Many studies pointed out that a minority of individuals at a high fracture risk actually receive treatment [116,117]. In this regard, Rodrigues et al. found that only 7.1% of 65-year-old women with fragility fractures were under treatment for osteoporosis, and 13.9% never had treatment [118].

A special need for drug therapy arises in patients who sustain a fragility fracture because of the increased risk of refracture [112]. However, it has been shown that the proportion of patients starting treatment to reduce the risk of future fracture within the year following a diagnosis of fracture is low [119]. In a study developed by the IOF, it was found that the treatment gap for the five largest EU countries (France, Germany, Italy, Spain and the UK), as well as Sweden, is estimated to be 73% for women and 63% for men. Moreover, in France, Sweden and Spain, 85%, 84% and 72% of fracture patients are without any treatment 1 year after fracture, respectively [120].

The treatment gap is particularly marked in the case of hip fracture patients [121,122]. Kim and colleagues studied the use of osteoporosis medications for the secondary prevention of osteoporotic fracture. Among a total of 86,202 patients with hip fracture, only 11 to 39% were treated with osteoporosis medication within 3 months after the fracture. Moreover, the adherence to osteoporosis treatment was also suboptimal [122].

One of the challenges in the treatment of patients with osteoporosis is that, in clinical practice, it is often the case that they show poor compliance with the pharmacotherapy that is prescribed to them. Among the reasons underpinning the low adherence to pharmacotherapy in patients with this pathology is that the asymptomatic nature of the disease maintenance treatment means that the patient sees no manifest benefit from the treatment [112]. Some reports found that the use of telecarers has the potential to be a useful adjunct in the monitoring of osteoporosis treatment and compliance. However, the participation of patients, families, physicians and clinicians is critically important for the success of this tool [123].

Other explanatory factors for the low adherence to pharmacotherapy among osteoporosis patients are: the cost of the drug, concerns regarding the long-term efficacy of the osteoporosis treatment and the fear of rare side effects. Long-term treatment with bisphosphonates is generally well tolerated for the patient and is considered safe and effective. However, the most important reason why patients decide to stop oral forms of bisphosphonates treatment is gastrointestinal irritation. In the most severe cases, reactions in the upper gastrointestinal tract may also include esophageal erosion or esophageal ulcers [124]. Moreover, bisphosphonates treatment has been shown to be associated with the occurrence of rare side effects which include osteonecrosis of the jaw (ONJ), atypical femoral fractures and cardiovascular damage [112].

ONJ is a serious but rare effect of antiresorptive agents or angiogenesis inhibitors. This pathology is characterized by progressive bone destruction in the maxillofacial area of patients without previous radiation therapy or metastatic disease in the jaws [125]. The risk of bisphosphonate-associated ONJ in patients with long-term therapy has been estimated to be 0.21% over four years of therapy [126]. Recently, it has been reported that the risk of ONJ is higher in patients receiving denosumab therapy compared with those receiving bisphosphonates. In this study, among 9956 registered patients who underwent at least one dual-energy X-ray absorptiometry examination, 17 cases of ONJ were identified. Of these 17 patients, 12 were receiving denosumab at the time of ONJ diagnosis, and 5 of the patients were treated with oral or intravenous bisphosphonate therapy [127]. Interestingly, it has been highlighted that dental management by the screening and treatment of oral diseases during and also after the treatment with ONJ-related drugs can significantly reduce the occurrence of this disease [128].

Atypical femoral fractures are unusual fractures along the femoral diaphyseal and are located in the subtrochanteric region, which occur with little trauma. In a study aimed to evaluate the risk of atypical femur fracture in nearly 200,000 women using bisphosphonates over a ten-year period, the risk of atypical femur fracture increased with longer durations of bisphosphonate use and rapidly decreased after bisphosphonate discontinuation [129]. Although drug holidays decrease the risk of atypical femur fracture, the effect of discontinuation on other osteoporotic fractures must also be considered. The same study also found that, after 3 years of bisphosphonates treatment, 149 hip fractures were prevented, and 2 bisphosphonate-associated atypical fractures occurred in White women. Among Asian women, the balance after 3 years was 8 bisphosphonate-associated atypical fractures compared with 91 hip fractures prevented. By 10 years, bisphosphonates treatment decreased the risk of osteoporotic and hip fractures and outweighed the increased risk of atypical fractures among White women (less so among Asian women) [129].

Additionally, adverse effects have been observed with the use of romosozumab. The treatment with this anti-sclerostin antibody might increase cardiovascular damage and therefore the risk of cardiovascular complications such as cardiac ischemic or cerebrovascular events [130].

In summary, the careful evaluation of patients’ previous disorders and the consideration of correct timing, age and comorbidities are necessary to evaluate the risk–benefit ratio of a specific drug therapy. Nevertheless, in the worst scenario, an alternative treatment option is often available. Moreover, for the reduction of the treatment gap, it is suggested that the international development of the Fracture Liaison Services (FLS) should be carried out to better identify patients who are at a high risk of fractures. Moreover, FLS provides an opportunity to improve the adherence to treatment, with a consequent reduction in the risk of refracture [131,132].

### 4.3. Cell Therapy as a Novel Approach

Osteoporosis is a disease characterized by a change in cell differentiation, with osteogenic differentiation inhibited in favor of adipogenic differentiation [133]. Aging increases the likelihood of osteoporosis, as older patients produce fewer stem cells with self-renewal and immunomodulatory capacities [134]. Clinical therapies for the treatment of osteoporosis have so far focused on bone remodeling and preventing bone loss, although they are not fully effective [57,61].

Cell therapies have attracted great interest in recent years for the treatment of certain chronic diseases including osteoporosis. This type of therapy focuses on the ability of cells to repair damaged tissue [135]. Several preclinical studies have focused on the use of stem cells of different origins [136].

MSCs from bone marrow or adipose tissue are the cells considered optimal for this type of treatment, as they are immunoprivileged and immunomodulatory cells, and their use is approved by the FDA. However, embryonic stem cells or induced pluripotent stem cells have been discarded due to ethical and safety concerns [137].

The results showed that, by using a high number of progenitor stem cells with good proliferation and differentiation capacities, it is possible to control bone resorption, decrease fracture damage and improve tissue mineral density in the treatment of osteoporosis [138].

After transplantation, MSCs may contribute to bone formation through two possible mechanisms of action. On the one hand, MSCs target the damaged site and differentiate into osteogenic cells, and on the other hand, MSCs secrete characteristic growth factors, such as the vascular endothelial growth factor (VEGF), transforming growth factor beta (TGF-β), hepatocyte growth factor (HGF) or insulin-like growth factor-1 (IGF-1), which act by promoting bone remodeling processes and preventing bone loss [139].

Decades ago, clinical studies were already conducted in children, using bone marrow MSCs (BM-MSCs) for the treatment of severe osteogenesis imperfecta [140].

For cell delivery, MSCs can be administered systemically (intravenous or intra-arterial injection) or locally (intracoronary or direct injection into damaged tissue). The cell migration procedure is not yet fully understood, so it is difficult to determine which mode of application is the most beneficial. It would be interesting to monitor the injected cells and their ability to adhere to the damaged tissue to determine this [141].

In a recent study by Lu et al., it was observed that extracellular vesicles (EVs) from MSCs possessed therapeutic potential for the treatment of osteoporosis, similar to that of progenitor cells. To develop this idea, they focused on studying disease models, potential therapeutic targets and the molecular mechanisms of action. The use of EVs in osteoporosis has not yet been studied, but it is being studied in the treatment of cancer, renal and cardiovascular diseases and wound healing. Furthermore, the great advantage of this type of treatment is that it is completely cell-free, eliminating any possibility of rejection in the patient. This would facilitate its application in the clinic [142].

Genetically modified BM-MSCs have also been studied in the treatment of osteoporosis. Sui et al. demonstrated that this cell line showed good homing and osteogenic capacity in glucocorticoid-induced murine osteoporosis (GIOP). The transplantation of allogeneic BM-MSCs reduced the loss of bone tissue mass and hardness and promoted osteoblastogenesis while maintaining bone formation [143].

To regulate osteoblast differentiation, two very important transcription factors, Runx2 and osterix, must be taken into account. The activation or inhibition of these transcription factors controls osteogenic differentiation in MSCs. In addition, miRNA regulators are also important, as they have a suppressive effect on bone cell formation while promoting adipocyte formation. Physical and chemical factors also affect proper bone remodeling and formation and may be of help when it comes to treatment [144].

Even so, it is necessary to continue adapting this type of therapy, as there is the problem of controlling cell migration once the MSCs have been implanted. The cells themselves do not recognize the bone surfaces to be treated, which can trigger cell differentiation to another, non-osteogenic type, and this type of graft is not able to be maintained in the long term [145].

Currently, there are few clinical trials studying the effect of infusion cell therapy for the treatment of osteoporosis. ClinicalTrials.gov was searched for the most recent clinical advances in the field. A search was carried out including the terms “Osteoporosis” and “Cell therapy”. Four clinical trials have been found in which cells were directly applied with therapeutic applications in osteoporosis (Table 2). Among the different cell types used, we can find MSCs (NCT04501354), fucosylated MSCs (NCT02566655), allogeneic adult umbilical cord-derived MSCs (NCT05152381) and autologous osteoblastic cells (NCT02061995). As of yet, no results have been reported for these trials.

### 4.4. Hydrogels for Osteoporosis Treatment

In the post-traumatic bone tissue repair process, autologous/allogeneic transplantation has so far been the main path followed by experts, but it presents a major problem of donor limitation. New applications of different biomaterials are presented as a real alternative in bone regeneration. The use of biomaterials in the hydrogel state stands out due to their hydrophilic properties, their good biocompatibility, their porous structure and their adjustable biodegradability mechanical properties. These properties directly influence the cell migration, proliferation and differentiation of MSCs, which favors bone regeneration [146]. Hydrogels have been used as an emerging and promising tool in tissue engineering. They act as a substitute for the conventional materials used in restorative surgery by combining biology and engineering, improving and restoring tissue function [147].

One of the current challenges in tissue engineering for the treatment of osteoporosis is the development of a system for the controlled release of therapeutic substances that can improve their targeting. Injectable hydrogels are presented as a versatile option for different applications in tissue engineering thanks to their adaptability. Despite this, their clinical application is still scarce, and more studies are required to improve the aspects related to the use of polymeric biomaterials, their mechanical properties or their biodegradability [148]. Zheng et al. analyzed different strategies based on hydrogels for the treatment of osteoporosis, concluding that the use of biomaterials based on combined natural and synthetic composites is the best therapeutic strategy. These hydrogels have low cytotoxicity and good biocompatibility and biodegradability, which, together with a physicochemical crosslinking process, improve the mechanical properties of the construct. This makes it possible to control the degradation rate of the hydrogel, generating an excellent vehicle for the controlled release of drugs [149].

Recombinant human BMP-2 was, until a few years ago, the only osteoinductive growth factor approved by the FDA and the European Medicines Agency (EMA) for the treatment of long fractures [150]. However, the direct use of BMPs has been reported to lead to adverse effects, so the use of drug carriers is suggested as an option to reduce the doses applied and improve their cost-effectiveness [151].

Echave et al. developed an osteoconductive hydrogel based on gelatin and calcium sulfate-hydroxyapatite bioceramics that slowed the delivery of the required doses of growth factors such as BMP-2 to promote bone regeneration in an osteoporotic defect model. The resulting hydrogels were biocompatible and had an increased pore size, which favored mechanical compression properties. In this study, it was demonstrated that the hydrogels promoted the adhesion and proliferation of human bone marrow-derived MSCs and also promoted the osteogenic differentiation of the cells [152].

García-García et al. developed two different scaffolds based on PLGA-Alginate in a hydrogel state (HY) and another in a solid-state as a sponge (SS), which were for the sustained delivery of β-estradiol and BMP-2 for bone regeneration in osteoporosis. In this case, both systems were flexible, adapted well to the shape of the defect and had the same controlled release rate of β-estradiol and BMP-2. According to their trials, both strategies promoted bone regeneration, but in the case of SS, the bone repair was 30% higher than that with HY. This was possible simply due to the shorter degradation time of SS compared to that of HY. This study reflects the importance of modifying the physical properties of hydrogels to optimize regenerative therapies [153]. In another similar study by the same group, a heat-resistant injectable hydrogel was used to encapsulate 17β-estradiol, bone morphogenetic protein-2 (BMP-2) and plasma rich in growth factors (PRGF) microspheres. Here, the loaded hydrogel was applied locally to regenerate a critical calvarial bone defect in rats. PRGF did not increase bone repair, while the addition of BMP-2 increased the response to 17β-estradiol. However, the mineralization of newly formed bone in the osteoporosis groups was markedly lower than that in the non-osteoporosis groups [154].

In the treatment of diseases mainly caused by osteoporosis, such as hypercalcemia, the use of hydrogels may present an advantage for the regulation of calcium formation [155]. Li et al. developed an injectable tetra-PEG-based hydrogel loaded with the drug alendronate (ALN), which allowed for the long-term controlled release of anti-osteoporotic molecules. These hydrogels effectively promoted bone regeneration at the implantation site in a minimally invasive manner [156].

Salmon calcitonin (sCT) is a product currently used in clinical regenerative medicine to regulate calcium metabolism in order to improve the treatment of disorders such as osteoporosis and hypercalcemia. As sCT in serum is rapidly cleared in vivo, Yu et al. designed a hydrogel based on the conjugation of sCT with oxidized calcium alginate (sCT-OCA) and hydroxypropyl chitin (HPCH). These gels were stable for up to 28 days and showed higher biocompatibility when used on pre-osteoblastic cells than sCT alone. In sCT-OCA, the activity of some osteogenic markers such as ALP increased by up to 63%, and calcium deposition increased by 42%, enhancing osteogenic cell differentiation [157].

In osteoporosis, excessive oxidative stress causes osteoblast and osteocyte apoptosis, leading to abnormal bone formation around the damaged area [158]. Melatonin is a hormone that has previously demonstrated its capacity for cell differentiation and bone remodeling and its usefulness in curbing excessive oxidative stress [159]. In a study by Xiao et al., a hydrogel with a dressing function based on methacrylate gelatin (GelMA) doped with melatonin for controlled and targeted release was developed. In a trial with MC3T3-E1 cells, it was shown that melatonin in controlled doses reduced the apoptosis caused by hydrogen peroxide-induced oxidative stress and restored the osteogenic potential of the cells. In addition, it increased the bone mass around the implant in ovariectomized rats treated with this adhesive [160].

On the other hand, Zhao et al. generated a bio-inspired mineralized hydrogel from the supramolecular assembly of nano-hydroxyapatite, sodium carbonate and polyacrylic acid (CHAp-PAA). These hydrogels proved to be able to maintain their morphology and mechanical properties. They were biocompatible, bioactive and osteoconductive in studies carried out using bone marrow mesenchymal stem cells. The results presented in this work demonstrated that these hydrogels enhanced bone growth by accelerating bone formation without the need for additional therapeutic agents [49].

Another study developed a nanoemulsion drug delivery system based on a fluvastatin hydrogel, using carbopol940 as a gelling agent. The drugs were intended to be administered transdermally and were subsequently evaluated for their anti-osteoporotic potential. The in vivo anti-osteoporotic results carried out in this research showed the formation of new bone in the trabecular region of osteoporotic rat femurs and an increase in load-bearing with respect to the damaged tissue [161].

The encapsulation of alendronate, a bone resorption inhibitor, in different chitosan-based hydrogels crosslinked using genipin (CS/bGP) for the prolonged local delivery of alendronate by injection is an aspect that could be of interest in the treatment of OP. Increasing the concentration of alendronate resulted in hydrogels with a lower porosity and higher density. The CS/bGP hydrogel ensured the controlled release of alendronate for an average of 50 days depending on the initial inhibitor load added, proved to be biocompatible and showed a low immunogenic response. In addition, alendronate-loaded hydrogel was shown to have a lower inflammatory response, higher cell proliferation and faster tissue maturation [162].

Papathanassiou et al. fabricated and characterized silica-based hydrogels for the purpose of releasing bis-phosphonates, which are a synthetic variant of pyrophosphates with advantageous bone remodeling properties. These hydrogels are injectable and thermosensitive and can be reused and refilled. In addition, by altering several factors, such as temperature, the cations present, pH and the structural characteristics of the bis-phosphonates, the release rate can be controlled [163].

Finally, in the literature, we can find studies that combine hydrogels with other types of physical strategies. Chen et al. studied the effect of the application of extracorporeal shock waves (ESW) together with the application of a hydrogel loaded with teriparatide (T-Gel), a drug used in the treatment of osteoporosis, on the activity and cell differentiation of osteoporosis-derived MSCs and their regenerative capacity. Their results showed that the combination of ESW and T-Gel significantly enhanced the viability, proliferation, migration and osteogenic differentiation of MSCs, thus improving the osteogenic activity of the microenvironment in osteoporotic defects [164].

### 4.5. Lifestyle and Osteoporosis

#### 4.5.1. Nutritional Habits

Calcium and vitamin D were previously considered to be the most important nutrients in preventing osteoporosis; however, there are other factors that can condition BMD and thus bone health [165]. Nutrients can have a direct or indirect influence on osteoporosis; direct influence is understood as being part of the bone structure itself, while indirect influence is related to the process of absorption and the utilization of calcium [166].

Without a doubt, when talking about nutrition and osteoporosis, calcium should be mentioned because it is one of the main constituents of bones and plays an important role in bone stiffness [167]. This nutrient plays an important role in osteogenesis by increasing the concentration of OPNand OC, promoting the formation of new bone and increasing the number of estrogenic markers such as ALP [168]. However, the calcium intake in the general population remains below that recommended by different organizations (<700 mg/day) [126], mainly due to the limited supply of calcium-rich foods [169]. Long periods of calcium deficiency lead to low BMD [170]; hence, the use of calcium supplementation has become very popular, with favorable effects in different populations and age groups [171]. Nevertheless, at present, controversy has arisen over the consumption of calcium supplements since they are associated with cardiovascular disease [172,173] and, when they are not administered with vitamin D, with the risk of myocardial infarction [174]. It is important to emphasize that these risks apparently do not occur when calcium intake comes from dietary sources, making these the best option [167].

Vitamin D is a steroid prohormone essential for the absorption and regulation of calcium in the intestine [170]. Vitamin D levels in the body depend mainly on subcutaneous production following the exposure to sunlight (80–90%) and, to a lesser extent, on diet (10–20%), due to the limited supply of vitamin D-rich foods [175]. This vitamin promotes adequate blood calcium levels, which promote bone growth and remodeling from osteoblasts and osteoclasts, decreasing the risk of osteoporosis [91]. However, some authors state that vitamin D supplementation alone has no effect on fracture risk [176,177] or BMD [178], attributing these results to the low calcium intake of the general population [179]. In contrast, Weaver et al. [91] conclude in their systematic review with a meta-analysis that joint vitamin D and calcium supplementation significantly decreases the risk of fracture in patients with and without osteoporosis.

Protein intake is an important nutritional factor for bone health, as it provides the amino acids necessary for the construction of the bone matrix and stimulates bone formation from IGF-I [180]. Cadogan et al. conducted a study in which they showed that a high consumption of milk (a protein-rich food) improved BMD and overall bone mineral acquisition. However, the evidence of the effects of protein intake on bone health is still weak [181,182,183], so further research is needed.

Soy isoflavones are considered to be the most estrogenic compounds found mainly in the legumes of the Fabaceae family [184]. These bioactive compounds have been deeply studied in recent years; it has been found that they have favorable effects on glucose levels [185], breast cancer risk [186] and osteoporosis [187], among others. As for the preventive effects of osteoporosis, these are better in postmenopausal women who have developed the pathology due to hormonal alterations, since they decrease osteoclastic factors such as collagen C-telopeptide and increase osteoblastic factors such as bone alkaline phosphatases [188] in addition to selectively antagonizing the catabolic action on the osteoblasts of parathohormones [189].

Theoretically, folic acid and vitamin B-12 could have important effects on fracture risk due to their action on homocysteine metabolism [170]. The consumption of folic acid and vitamin B-12 is expected to reduce the amount of homocysteine in the blood by a quarter or a third [190], which would slow down its degradative action on the extracellular matrix and decrease BMD [191]. However, the results showed that a chronic intake of folic acid and B-12 has no effect on the risk of fracture [192].

Finally, caloric intake is also an aspect to be considered. Restrictive diets are totally contraindicated in people with osteoporosis, since one of the risk factors for this pathology is thinness (<21 kg/m^2^ or <127 pounds). Another related aspect is the number of calories expended at rest at a neutral temperature defined as the Basal Metabolic Rate (BMR) [193]. BMR increases with the amount of cardiovascular exercise practiced and decreases with age. Hsu et al. [194], in their study, found that a BMR above 1182.7 Kcal is associated with better BMD in postmenopausal women, which translates into a lower risk of osteoporosis.

#### 4.5.2. Physical Exercise

It is clear that physical inactivity leads to a decrease in BMD, while physical exercise increases it [170]. BMD depends on the dynamic balance between bone formation and resorption [195], with mechanical loads being the main stimulus for osteoblastic differentiation and mineralization, promoting adequate bone mass and density [196]. In addition, physical exercise has an important hormonal effect by regulating estrogen, PTH and glucocorticoid levels, which are involved in bone metabolism [195]. The constant practice of physical exercise promotes the proliferation of estrogens [197], which are bone protectors since they slow down the production of osteoclastic cytokines, favor the proliferation of osteoblasts and decrease osteocyte apoptosis [198].

Another effect of physical activity related to osteoporosis is related to BMR; cardiovascular and resistance exercise have been shown to increase BMR levels [199]. Several authors have tried to estimate BMD through different methods such as anthropometric measures; however, measures such as the waist hip index are insufficient to achieve a reliable estimate of BMD, while BMR could be positioned as an important predictor of osteoporosis because it has a direct relationship with BMD—the higher the BMR, the better the bone health that is expected [194].

The usual practice of physical exercise has an important effect on body weight, which is closely related to BMD, the most important component of body composition, and lean mass for its effects on BMD in the whole body, while fat mass has only been related to femoral neck BMD [200].

The American College of Sport Medicine recommends, for the prevention or treatment of osteoporosis, that, in addition to the minimum physical activity established by the WHO, weight-bearing exercise should be performed whenever bone loss is mild or is to be prevented; however, when BMD has been compromised, the added stress to the bones may represent an increase in the risk of fracture [201]. There is still no clear consensus on the ideal exercise prescription for this pathology; however, Howe et al. [202], through a systematic review, analyzed 43 RCTs and found that the best exercises to improve BMD in the femoral neck were high strength exercises without weight bearing, while for the spine, combinations of exercises were better. On the other hand, Kemmler et al. [203], through a systematic review with a meta-analysis, concludes that, although positive effects of exercise on fracture risk in old age were found, they were weak. This is mainly due to the wide variety of exercises that can generate different effects.

Some of the most commonly used training modalities in patients with osteoporosis are resistance exercise, aquatic exercise and proprioceptive training [204]. On the one hand, resistance exercise seems to act on the bone from the myotendinous junction, where the increase in tendon tension resulting from muscle contraction stimulates the osteogenic response of the bone, increasing BMD [205]. On the other hand, aquatic exercises, despite not being the best option to increase BMD due to their low or even null impact on bone, do allow for the generation of muscle tension while minimizing any risk of falling, which, for older adults, is a clear advantage [206]. Previously, it was believed that aquatic exercises decreased BMD; however, studies such as that by Su, Chen and Xie [207] show the opposite. Although these types of exercises are not the best for increasing BMD, they do have positive effects on it. Finally, proprioceptive exercises are also one of the main strategies to address the osteoporotic population; the improvement in the perception of the location of their own body in space from exercise has been shown to decrease the risk of falls and increase mobility, in addition to improving functional capacity and dynamic balance, resulting in an improvement in the quality of life of these patients [208].

#### 4.5.3. Alcohol Intake and Smoking

Alcohol consumption has shown heterogeneous effects depending on the dosage. When alcohol is consumed in light or moderate doses, it functions as a protective factor for BMD, while when consumption is high, it is consolidated as a risk factor for fracture [209]. Different studies have shown positive effects of alcohol consumption in low amounts. Berg et al. determined that when a person consumes between 0.5 and 1.0 drinks per day, they have a lower risk of hip fracture [210]. On the other hand, lifestyle habits do not seem to be related to the protective effect of moderate alcohol consumption in women close to menopause [211]. The protective effect of alcohol on bone health could be explained by its acute suppressive effect on bone resorption without the participation of PTH or calcitonin [212], as evidenced by the low levels of CTX associated with ethanol intake [213], while high alcohol consumption interferes with the calcium balance by decreasing its absorption in the intestine, reducing vitamin D production and increasing the risk of falls [210].

On the other hand, in the 1980s, cigarette smoking was identified as a risk factor for osteoporosis [214], which, to date, remains prevalent and increasing, mainly because of the addictive nature of this habit [215]. Different authors have evidenced a negative and independent relationship between cigarette smoking and bone health [216]. In older adults, it has been shown that smoking generates an increase in the loss of bone mass and the risk of fracture [217], which can be explained by the free radicals produced by the consumption of about 150 toxins in cigarettes, leading to an increase in estrogen-destroying enzymes, which, as previously mentioned, are hormones of great importance for the process of bone remodeling [209]. This also explains the early onset of menopause in female smokers, with the consequent effects of this condition on bone health [218]. However, it is unclear which of the toxins found in cigarettes are specifically related to the alteration of bone health [219].

Chronic cigarette smoking suppresses the production of OPG, a protein that works as an inhibitor of osteoclastogenesis, which results in an increase in the number of osteoclasts, thus favoring bone resorption processes [220]. Tang and Lappin, in their articles, studied this phenomenon, finding that individuals who smoked cigarettes presented a lower level of OPG, while the RANKL (receptor activator of nuclear factor κβ ligand), which works as a stimulant of osteoclastic maturation and activity, was higher than those found in non-smoking subjects [221,222]. Smoking also induces inflammatory processes and increases oxidative stress, generating damage to the collagen metabolism, which acts as an important biochemical marker in bone metabolism in addition to inducing toxicity on bone cells by increasing the resistance to calcitonin, which blocks bone angiogenesis [223], preventing the creation of new blood vessels, which would alter the flow of oxygen and nutrients to the bone [224]. In addition to the inverse association between smoking and body weight described in the evidence, it has been shown that cigarette consumption is associated with low weight due to the inhibitory effect of nicotine on appetite [219].

Additionally, it has been observed that the risk of falls increases directly with cigarette smoking. Ampelas, in his systematic review, concludes that the risk of osteoporosis and hip fracture increases due to cigarette smoking because of its negative effect on BMD, regardless of the sex of the subject [225]. Finally, it has been shown that smoking cessation produces an increase in BMD, which reduces the risk of fracture [209]; however, these effects are only appreciable after 10 years of non-consumption [225].

## 5. Conclusions

Osteoporosis is a severe, chronic, progressive and clinically silent disease which results from an imbalance between bone resorption and bone production. Osteoporosis does not follow pre-established clinical patterns but rather manifests with specific signs and symptoms during its course, including pain, deformities or a loss of height. Fragility fractures are the most common consequence of osteoporosis and are particularly common in the vertebrae, hip and forearm. Despite advances in the diagnosis through different methods such as bone densitometry and dual X-rays, more research is needed.

Current FDA-approved osteoporosis treatments mainly consist of the use of drugs designed to decrease bone resorption. A better understanding of the markers, cellular events and genetic targets of osteoporosis has contributed to the development of novel drug agents. Thus, new targets are being studied for the treatment of osteoporosis, such as cathepsin K inhibitors or anti-sclerostin therapies; however, an ideal osteoporosis therapy has not yet been developed, as they still present considerable adverse effects that limit their long-term use. To overcome these problems, regenerative medicine is now an area of intensive exploration. Among new therapeutic strategies, MSCs are expected to be a promising tool due their immune-privileged potential and their role in bone repair. After transplantation, MSCs may contribute to bone formation through two possible mechanisms of action: by their ability to graft into tissues and differentiate into osteogenic cells, or by secreting characteristic growth factors that promote bone remodeling processes and prevent loss. Moreover, clinical trials using MSCs as the principal treatment are underway. The future evaluation of these studies will provide us with information about the safety, tolerability and efficacy of the transplanted cells and will open the door to establish their therapeutic mechanism in osteoporosis. Meanwhile, other techniques, such as gene modification, the use of EVs and the combination of cells and hydrogels, are under study to improve the activity of stem cells that may represent novel therapeutic approaches in future clinical practice.

In addition, there are non-pharmacological methods for the prevention of further osteoporotic fractures and for the regulation of osteoporosis which are related to lifestyle factors. Critical lifestyle factors include nutritional habits such as maintaining adequate calcium and vitamin D intake, engaging in regular weight-bearing physical activity and avoiding excessive alcohol intake and smoking.

## Figures and Tables

**Table 1:** Drugs for the treatment of osteoporosis.

	Drug Names	Description	Indication
Anti-Resorptive			
Selective oestrogen-receptor modulators	Raloxifene	They act as estrogen receptor agonists, thereby decreasing bone resorption.	- Postmenopausal OP - Postmenopausal OP with a high fracture risk
	Bazedoxifene		
Calcitonin		Their main function is to prevent the loss of bone mass due to sudden immobilization.	- Immobilizations
Bisphosphonates	Alendronate	They are the first choice in postmenopausal osteoporosis. They act by binding to the bone and preventing bone resorption.	- Postmenopausal OP - Postmenopausal OP with a high fracture risk - Advanced neoplasia with bone involvement and tumor-induced hypercalcemia
	Risedronate		
	Ibandronate		
	Zoledronic acid		
RANKL antibody	Denosumab	Human IgG2 monoclonal antibody that has a high specificity and affinity for RANKL, which it binds and inhibits.	- Advanced neoplasia with bone involvement - Treatment of giant cell tumors of unresectable bone or when surgical resection involves severe morbidity
Anabolic Agents			
Parathyroid hormone analogs	Teriparatide	Increases bone formation with minor increases in bone resorption, resulting in a net anabolic effect.	- Postmenopausal OP and men at a high fracture risk - OP associated with glucocorticoid treatment in women and men at a high fracture risk
	Abaloparatide		

OP: Osteoporosis.

**Table 2:** Clinical trials with cell therapies for osteoporosis.

Cell Type	NTC Number	Title	Phase	Indication
MSC	NCT04501354	Evaluation of Clinical and Bone Density Improvement After Implantation of Allogenic Mesenchymal Stem Cell From Umbilical Cord on Osteoporosis Patients	2	Improvement of bone mass density
Fucosylated MSC	NCT02566655	Clinical Trial of Intravenous Infusion of Fucosylated Bone Marrow Mesenchyme Cells in Patients with Osteoporosis (CSM/OP/2011)	1	Osteoporotic low-impact fractures
Allogeneic adult umbilical cord-derived mesenchymal stem cells	NCT05152381	Safety of Cultured Allogeneic Adult Umbilical Cord Derived Mesenchymal Stem Cell Intravenous Infusion for Osteoporosis	1	OP
Autologous osteoblastic cells	NCT02061995	Phase 2a Study on Intravenous Infusion of Autologous Osteoblastic Cells in Severe Osteoporosis	2	Severe OP

OP: Osteoporosis.

## Abbreviations

MSCs: mesenchymal stem cells; BMD: bone mineral density; ALP: alkaline phosphatase; OPN: osteopontin; OC: osteocalcin; PTH: parathyroid hormone; TGF-β: transforming growth factor beta; M-CSF: macrophage colony-stimulating factor; RANKL: receptor activators of nuclear factor-κβ ligand; OPG: osteoprotegerin; LPL: lipoprotein-related co-receptor; IOF: International Osteoporosis Foundation; BMTs: bone turnover markers; Pir: Pyridinolines; Dpir: deoxypyridinoline; ICPT: C-terminal telopeptide of type I collagen; β-CTX: β-CrossLaps; NTX: N-terminal telopeptide of type I collagen; FDA: Food and Drug Administration; SERMs: selective oestrogen receptor modulators; ER: estrogen receptor; FPPS: farnesyl pyrophosphate synthase; ONJ: osteonecrosis of the jaw; FLS: Fracture Liaison Services; VEGF: vascular endothelial growth factor; TFG- β: transforming growth factor beta; HGF: hepatocyte growth factor; IGF-1: the insulin-like growth factor-1; BM-MSCs: using bone marrow mesenchymal stem cells; EVs: extracellular vesicles; GIOP: glucocorticoid-induced murine osteoporosis; EMA: European Medicines Agency; HY: hydrogel state; SS: solid-state; PRGF: plasma rich in growth factors; ALN: alendronate; sCT: salmon calcitonin; sCT-OCA: salmon calcitonin with oxidized calcium alginate; GelMA: methacrylate gelatin; CHAp-PAA: nano-hydroxyapatite, sodium carbonate and polyacrylic acid; ESW: extracorporeal shock waves; T-Gel: teriparatide; BMR: Basal Metabolic Rate.
